# U-shaped association of serum magnesium with mild cognitive impairment among hemodialysis patients: a multicenter study

**DOI:** 10.1080/0886022X.2023.2231084

**Published:** 2023-07-10

**Authors:** Yuqi Yang, Yanjun Long, Jing Yuan, Yan Zha

**Affiliations:** Department of Nephrology, Guizhou Provincial People’s Hospital, Guiyang, Guizhou, China

**Keywords:** Magnesium, mild cognitive impairment, *U*-shaped relationship, hemodialysis

## Abstract

**Background:**

The optimal serum magnesium level of patients undergoing hemodialysis (HD) with cognitive impairment is still unclear. This study aimed to evaluate the association between serum magnesium levels and mild cognitive impairment among HD patients.

**Methods:**

This was a multicenter observational study. Patients undergoing hemodialysis from 22 dialysis centers in Guizhou Province, China were recruited into the study. HD patients were divided into five groups according to serum magnesium quintile. Cognitive function was measured with Mini Mental State Examination. The outcome was an incident mild cognitive impairment (MCI). Multivariate logistic regression analysis, restricted cubic spline and subgroup analysis were applied to explore the association of serum magnesium level with MCI.

**Results:**

Among 3562 HD patients (mean age 54.3 years, 60.1% male), the prevalence of MCI was 27.2%. After adjusting for confounders, serum magnesium 0.41–0.83 mmol/L [odds ratios (OR) 1.55, 95% confidence interval (CI): 1.10–2.18] had a higher risk of MCI compared with serum magnesium of 1.19–1.45 mmol/L. A U-shaped association was identified between the serum magnesium and incident MCI (P for non-linearity = 0.004). The optimal range of magnesium level with the lowest risk of MCI was 1.12–1.24 mmol/L. As the serum magnesium level was lower than 1.12 mmol/L, the risk of MCI decreased by 24% per standard deviation (SD) increase in serum magnesium (OR 0.76, 95%CI: 0.62–0.93); when the serum magnesium level exceeds 1.24 mmol/L, a rise per SD increased the risk of MCI by 21% (OR = 1.20, 95%CI: 1.02–1.43). Subgroup analyses demonstrated that the associations were robust among individuals with low educational level, smoking, living alone, no working, and without hypertension or diabetes.

**Conclusions:**

Serum magnesium has a U-shaped association with MCI among HD patients. Both lower and higher serum magnesium can increase the risk of MCI for this population specifically. The optimal serum magnesium range with the lowest risk of MCI was 1.12–1.24 mmol/L.

## Introduction

Mild cognitive impairment (MCI) is common in patients with chronic kidney disease [1], especially in hemodialysis (HD) patients with end-stage kidney disease (ESKD) [2]. The prevalence of cognitive impairment ranges from 70–80%, which is approximately up to three times higher than that in the age-matched general population [3–5]. MCI is closely associated with an increased risk of compromised quality of life and functional capacity, as well as adverse outcomes, including hospitalization, and mortality [6,7]. Currently, there are no effective pharmacologic therapies to target and affect the process of cognitive impairment. Identification of modifiable risk factors may offer novel strategies to prevent MCI among HD patients.

Magnesium, the second most abundant intracellular cation in the human body, plays a crucial role in various biological processes, including energy metabolism, glycolysis, protein, and nucleic acid synthesis [8]. Abnormal serum magnesium level is common in HD patients. Previous studies have shown associations of magnesium and several clinical outcomes, including mortality, cardiovascular events, and hospitalization [9,10]. Additionally, magnesium is essential in the regulation of excitatory synaptic transmission, neuronal plasticity, and neuronal protection [11], which implies that magnesium may have a potential role in neurological disorders, including cognitive impairment.

To date, there is disagreement about the exact nature of magnesium and cognitive impairment. The majority of previous studies have mainly focused on hypomagnesemia and cognitive impairment. Several studies have demonstrated low serum magnesium as a risk factor for cognitive impairment in the general population, however, the results of individual studies are inconsistent due to the discrepancies in study design, study population, sample size, and assessments of magnesium, cognitive function [12–14]. Few studies have explored the association between hypermagnesemia and cognitive impairment [15].

To our best knowledge, there is little evidence about the relationship between serum magnesium and cognitive impairment among HD patients. With the goal of growing our understanding on the role of magnesium in cognitive impairment, we aimed to assess the association of serum magnesium with incident MCI in HD patients with a large-scale, multicenter HD cohort.

## Materials and methods

### Study population and setting

**Figure 1.**
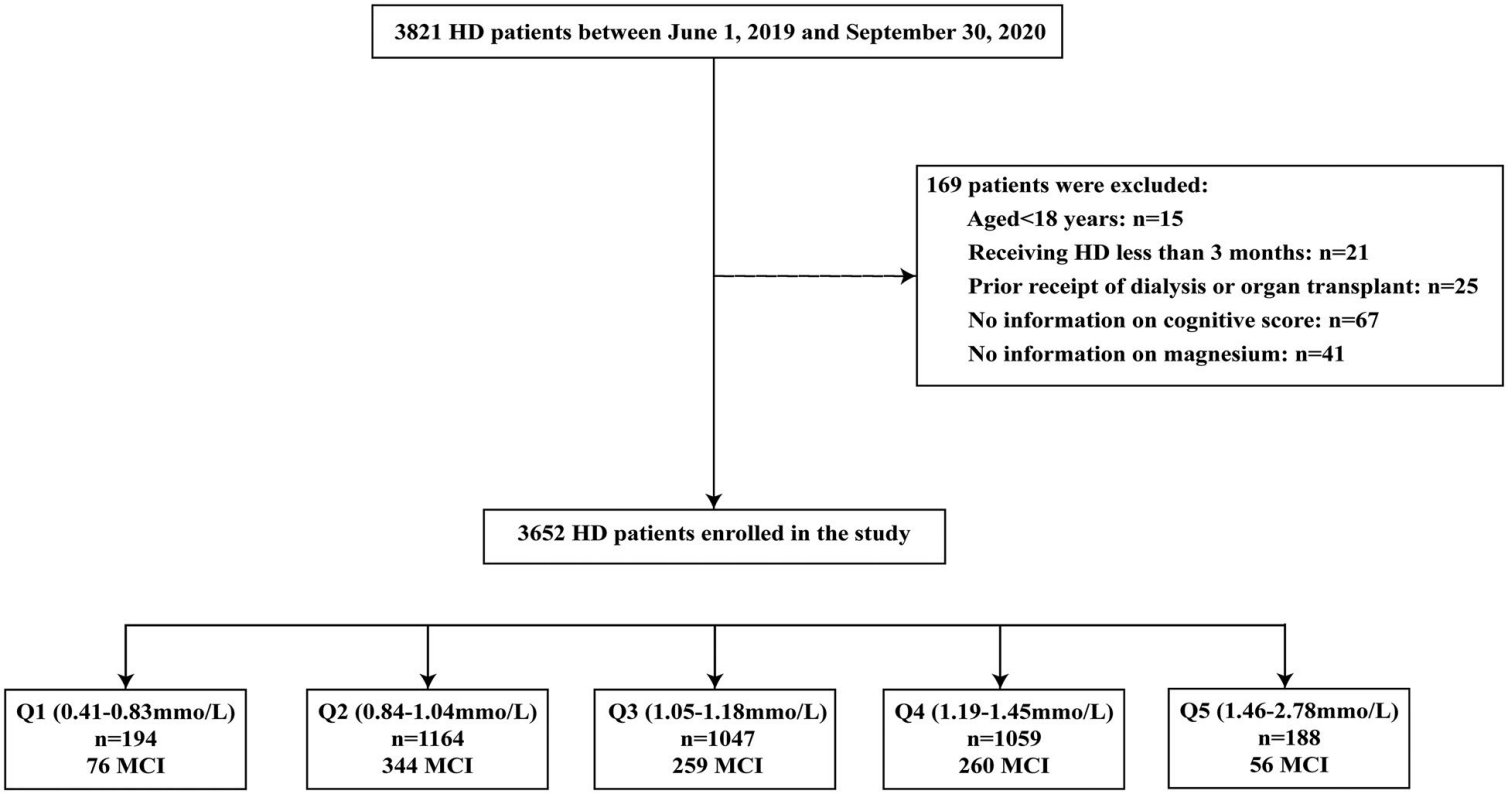
**Flow chat of the study.** HD: hemodialysis; MCI: mild cognitive impairment.

This was a multicenter, observational cohort study, and recruited the patients undergoing maintenance HD from 22 HD centers in Guizhou Province, China between 1 June 2019 and 30 September 2020. Patients were eligible for inclusion if aged ≥ 18 years old, receiving maintenance HD for at least three months, and had completed the biochemical measurements, and questionnaire records. Our analysis excluded individuals with prior receipt of dialysis or organ transplant, with severe mood disorders or psychotic disorders, and missing magnesium and cognition measurements. More details on the inclusion process of studied populations were provided in Figure 1. All the patients performed HD with conventional dialyzers under the standard temperature (35.5–36.5 °C). The dialysate composition is usually composed of sodium (130–140mmol/L), potassium (3-4mmol/L), chloride (96–110mmol/L), calcium (1.5–1.75 mmol/L), magnesium (0.6–1.0 mmol/L), bicarbonate (32–38mmol/L). The electrolyte concentrations would be adjusted accordingly.

A professional team composed of eight to ten dialysis doctors was dispatched to each HD center. Each doctor has been trained uniformly for assessing cognitive impairment and conducting the questionnaire, in order to maintain consistency of the study. All the enrolled patients were informed and signed written informed consent. The study was approved by the Ethics and Research Committee of Guizhou Provincial People’s Hospital (Approval number: [2020]208) and adhered to the Declaration of Helsinki and subsequent amendments.

### Outcome variable: cognitive function

Cognitive function was assessed using the Mini-Mental State Examination (MMSE) questionnaire by professional dialysis doctors at one hour of dialysis treatment, in order to eliminate the influence of HD treatment [16]. It assessed cognitive function in 5 components: orientation (5 points for temporal orientation, 5 points for spatial orientation), memory (3 points for immediate recall, 3 points for delayed recall), serial subtraction (5 points), language ability (2 points for naming, 3 points for oral command comprehension, 1 point each for repetition, reading and writing), and visuospatial ability (1 point) in order. The scores range from 0 to 30 points, with higher scores denoting better cognitive function. A score of 30–27 points means no cognitive dysfunction, A score < 27 on the MMSE can be diagnosed as MCI [17].

### Exposure variable: serum magnesium

Serum magnesium was measured in mmol/L by an automated colorimetric method, using the fast blood sample drawn in the morning before the dialysis treatment. All the blood collection was performed before MMSE testing for each patient. Based on the 5th, 35th, 65th, and 95th centiles of serum magnesium level (mmol/L), the enrolled patients were categorized as five groups: Q1 (0.41–0.83), Q2 (0.84–1.04), Q3(1.05–1.18), Q4 (1.19–1.45) and Q5 (1.46–2.78). The Q4 was used as the reference group in separate models.

### Covariate assessment

A well-designed general questionnaire was conducted by trained interviewers to collect sociodemographic characteristics, lifestyle behaviors, disease characteristics, and laboratorial measurements: age, sex, educational levels (high: >12th grade; low: <12th grade), smoking (yes or no), drinking (yes or no), working status (yes or no), and living status (living with partner or living alone); primary diseases of ESKD, dialysis vintages, vascular access (arteriovenous fistula, AVF or other access), the presence of hypertension (diagnosed as systolic blood pressure >140 mmHg and/or diastolic blood pressure >90 mmHg, or self-reported, or a medical record of responding diagnosis or medication; yes or no) and diabetes mellitus (diagnosed as HbA1c ≥ 6.5%, random blood glucose ≥ 11.1 mmol/L, fasting blood glucose ≥ 7.0 mmol/L, or self-reported, or a medical record of responding diagnosis or medication; yes or no), cerebrovascular disease (CVD; self-reported, or a medical record of responding diagnosis; yes or no); mean arterial pressure, body mass index (calculated as weight in kilograms divided by the square of height in meters, BMI, kg/m^2^), waist circumference, hip circumference; hemoglobin (g/L), platelets (×10^9^/L), serum albumin (g/L), creatinine (umol/L), urid acid (mmol/L), potassium (mmol/L), calcium (mmol/L), calcium (mmol/L), sodium (mmol/L), intact parathyroid hormone (iPTH, ng/mL), high-sensitive C-reactive protein (hs-CRP, mg/L), total cholesterol (CHOL, mmol/L) and total triglycerides (TG, mmol/L).

#### Statistical analyses

Participant characteristics were described across serum magnesium categories. The normal distribution was tested using the Kolmogorov-Smirnov test. Normally distributed continuous variables were described as means and SDs, and non-normally distributed continuous variables were expressed as median and interquartile range. Categorical variables were expressed as counts (percentages). One-way ANOVA, or Kruskal-Wallis *H*-tests for continuous variables and chi-square tests for categorical variables were used for the comparison across serum magnesium categories.

The multivariate logistic regression models were used to identify independent covariates and to estimate the effect of serum magnesium level on the risk of MCI, with odds ratios (ORs) and confidence intervals (CIs). Variables achieving *p*-value < 0.05 were entered into multivariate analysis during stepwise iteration. In model 1, there was no adjustment. In model 2, we adjusted for age and sex. In model 3, we adjusted for age, sex, educational level, alcohol drinking, smoking, working status, living status, hypertension, mean arterial pressure, waist-hip circumference ratio, serum uric acid, iPTH, and hs-CRP levels.

Restricted cubic spline (RCS) models were used for nonlinear relationships of serum magnesium levels based on multivariate logistic regression models. The number of knots was set as 4 (0.05, 0.35, 0.65, 0.95) because 4 knots not only provide a sufficient fit for the model but are a good compromise between flexibility and overfitting [18]. The likelihood ratio test was used for the tests for nonlinearity. If the relationships were non-linear, the difference of relationships at the threshold was performed using two piecewise linear regression models. An additional turning point for serum magnesium was determined by curve fitting the cognitive impairment corresponding to the turning point in the graph, and the range between the two points was considered to be the threshold for low risk of MCI. The risk associated with cognitive impairment is reported with per standard deviation (SD) of continuous serum magnesium. Furthermore, the subgroup analysis was conducted by age (< 45 or ≥ 45 years), sex (male or female), education (low or high education), smoking (yes or no), living status (yes or no), working status (yes or no), hypertension (yes or no), diabetes (yes or no).

All statistical analyses were performed using the statistical package R (The R Foundation; http://ww.r-project.org; version 4.0.1). Two-tailed tests were used and the *P* value <0.05 was considered statistically different.

## Results

### Patient characteristics

Figure 1 presents the flow chart of patient screening in the study. A total of 3821 HD patients were enrolled, and after applying the exclusion criteria, the final analytic cohort included 3652 HD patients. Table 1 lists the characteristics of the patients categorized by serum magnesium centile subgroups according to the knots. In the overall population, the mean age of 54.3 ± 15.2 years, and 60.1% were male. The mean serum magnesium level was 1.12 ± 0.21 mmol/L. There were significant subgroup differences in age, sex, educational level, HD vintages, vascular access, diabetes mellitus, CHD, mean arterial pressure, waist circumference, hip circumference, hemoglobin, serum albumin, creatinine, uric acid, potassium, calcium, iPTH and hs-CRP levels (all *P* < 0.05). The mean MMSE score was 27.5 ± 3.5 and the overall incidence of MCI was 27.2%. The MCI of HD patients with the lowest magnesium level was 39.2%, which was the highest among the five categories.

**Table 1.** Characteristics of HD patients by serum magnesium quintile.

Characteristic	Quintile of Serum Magnesium (mmol/L)						*P*-value
	All patients	Q1 (0.41–0.83)	Q2 (0.84–1.04)	Q3 (1.05–1.18)	Q4 (1.19–1.45)	Q5 (1.46–2.78)	
No. of patients	3652	194	1164	1047	1059	188	
Age, years	54.3 ± 15.2	58.2 ± 15.6	56.1 ± 15.7	54.2 ± 15.4	52.3 ± 14.4	52.0 ± 13.2	<0.001
Male, n(%)	2196(60.1%)	121(62.4%)	700(60.0%)	664(63.4%)	606(57.3%)	105(55.9%)	0.040
Educational level, n(%)	1038(28.4%)	45(23.2%)	353(30.3%)	312(29.8%)	283(26.8%)	45(23.9%)	0.066
HD vintages, months	40.0(19.0,73.0)	36.0(15.0,73.0)	37.0(18.0,73.0)	41.0(20.0,74.0)	42.0(22.0,73.0)	49.5(30.0,74.0)	0.009
Vascular access, AVF, n(%)	3157(86.4%)	148(76.3%)	974(83.5%)	926(88.4%)	938(88.7%)	171(91.0%)	<0.001
Smoking, n(%)	821(22.5%)	39(20.1%)	241(20.7%)	263(25.1%)	232(21.9%)	46(24.5%)	0.107
Drinking, n(%)	173(4.7%)	12(6.2%)	52(4.5%)	50(4.8%)	56(5.3%)	3(1.6%)	0.203
Working, n(%)	232(6.4%)	9(4.6%)	68(5.8%)	70(6.7%)	72(6.8%)	13(6.9%)	0.707
Living alone, n(%)	2770(75.8%)	125(64.4%)	853(73.2%)	785(75.0%)	857(81.1%)	150(79.8%)	<0.001
Hypertension, n(%)	2827(77.4%)	143(73.7%)	894(76.7%)	814(77.7%)	834(78.9%)	142(75.5%)	0.452
Diabetes mellitus, n(%)	959(26.3%)	62(32.0%)	333(28.6%)	282(26.9%)	234(22.1%)	48(25.5%)	0.003
CVD, n(%)	299(8.2%)	24(12.4%)	112(9.6%)	85(8.1%)	67(6.3%)	11(5.9%)	0.008
MAP, mmHg	98.9 ± 14.1	95.9 ± 14.7	98.5 ± 14.4	99.2 ± 14.0	99.2 ± 13.9	99.2 ± 13.2	0.028
BMI, kg/m^2^	22.8 ± 4.0	22.8 ± 5.3	22.9 ± 5.3	23.0 ± 3.8	22.6 ± 4.2	22.6 ± 3.5	0.255
Waist circumference, cm	83.1 ± 10.9	82.9 ± 11.0	84.0 ± 10.8	83.5 ± 11.0	81.8 ± 10.9	82.7 ± 10.9	<0.001
Hip circumference, cm	89.6 ± 8.1	88.9 ± 8.4	90.1 ± 8.1	89.8 ± 8.2	88.9 ± 7.9	90.3 ± 8.2	0.002
White blood cell, ×10^9^/L	6.42 ± 2.12	6.41 ± 2.31	6.48 ± 2.34	6.40 ± 1.98	6.40 ± 1.94	6.26 ± 2.28	0.689
Hemoglobin, g/L	108.8 ± 20.4	97.3 ± 21.2	105.9 ± 20.3	110.1 ± 19.6	112.0 ± 19.7	112.8 ± 22.2	<0.001
Platelet, ×10^9^/L	178.0 ± 63.2	181.1 ± 66.5	174.6 ± 62.9	178.0 ± 62.6	180.2 ± 63.4	183.3 ± 62.6	0.172
Serum albumin, g/L	40.3 ± 4.3	37.3 ± 5.2	39.8 ± 4.6	40.5 ± 4.0	41.1 ± 3.9	40.7 ± 4.1	<0.001
Creatinine, umol/L	922.6 ± 316.2	603.8 ± 315.6	814.0 ± 311.6	956.1 ± 272.0	1036.3 ± 277.7	1098.6 ± 323.5	<0.001
Urid acid, umol/L	437.1 ± 117.6	347.1 ± 142.8	404.0 ± 122.3	449.4 ± 105.5	466.8 ± 100.5	500.0 ± 108.4	<0.001
Potassium, mmol/L	4.7 ± 0.8	4.1 ± 0.9	4.5 ± 0.7	4.7 ± 0.7	4.9 ± 0.8	5.2 ± 0.8	<0.001
Calcium, mmol/L	2.17 ± 0.26	2.07 ± 0.29	2.15 ± 0.26	2.18 ± 0.25	2.21 ± 0.25	2.21 ± 0.30	<0.001
Sodium, mmol/L	139.1 ± 4.0	139.4 ± 4.0	139.0 ± 3.9	139.1 ± 4.4	139.0 ± 4.0	138.9 ± 3.4	0.616
iPTH, ng/mL	321.7(150.5,583.3)	276.5(128.4,470.0)	307.8(136.8,529.1)	350.5(173.5,649.5)	319.9(144.2,593.5)	326.1(167.9,667.4)	<0.001
CRP, mg/L	2.40(1.25,5.93)	3.69(1.24,12.4)	2.67(1.39,7.60)	2.25(1.21,5.50)	2.14(1.24,4.88)	1.96(0.76,3.95)	<0.001
Total cholesterol, mmol/L	3.91 ± 0.96	3.79 ± 0.96	3.94 ± 1.01	3.88 ± 0.94	3.94 ± 0.90	3.94 ± 1.02	0.147
Triglycerides, mmol/L	1.49(1.03,2.31)	1.35(0.93,2.06)	1.53(1.07,2.37)	1.49(1.02,2.35)	1.49(1.04,2.23)	1.58(1.01,2.42)	0.057
MMSE	27.5 ± 3.5	26.7 ± 3.8	27.2 ± 3.8	27.7 ± 3.3	27.8 ± 3.3	27.1 ± 4.1	<0.001
MCI, n(%)	995(27.2%)	76(39.2%)	344(29.5%)	259(24.7%)	260(24.6%)	56(29.8%)	<0.001

Note: *P* < 0.05 was considered statistically significant. Values were expressed as mean ± SD, median (25th–75th percentile), or frequency (percentage) as appropriate. Abbreviations: HD: hemodialysis; AVF: arteriovenous fistula; CVD: cerebrovascular disease; MAP,:mean arterial pressure; BMI: body mass index; iPTH: intact parathyroid hormone; CRP: C-reactive protein; MMSE: mini mental state examination; MCI: mild cognitive impairment.

### Relationships of serum magnesium level with MCI

Serum magnesium level was associated with MCI. Compared with those with serum magnesium levels of 1.19–1.45 mmol/L as a reference, HD patients with serum magnesium levels of 0.41–0.83 mmol/L (OR = 1.55, 95%CI: 1.10–2.18) showed a higher risk of MCI, after adjusting for age, sex, educational level, smoking, working status, living status, hypertension, diabetes, CHD, mean arterial pressure, and biomedical parameters. While, no significant differences were found in the other groups (Table 2).

**Table 2.** Association between serum magnesium level and MCI among HD patients.

Serum magnesium (mmol/L)	Model 1		Model 2		Model 3	
	OR (95%CI)	*P*-value	OR (95%CI)	*P*-value	OR (95%CI)	*P*-value
**MCI**						
Q1 (0.41–0.83)	1.97 (1.43–2.72)	<0.001	1.79 (1.29–2.48)	<0.001	1.55 (1.10–2.18)	0.012
Q2 (0.84–1.04)	1.28 (1.06–1.55)	0.010	1.19 (0.98–1.44)	0.077	1.15 (0.94–1.40)	0.176
Q3 (1.05–1.18)	1.01 (0.83–1.28)	0.941	0.98 (0.80–1.20)	0.860	0.97 (0.79–1.19)	0.753
Q4 (1.19–1.45)	Reference		Reference		Reference	
Q5 (1.46–2.78)	1.30 (0.92–1.83)	0.133	1.32 (0.93–1.87)	0.118	1.32 (0.93–1.87)	0.126

Note: *P* < 0.05 was considered statistically significant. Abbreviations: HD: hemodialysis; OR: odds ratio; CI: confidence interval; MCI: mild cognitive impairment. Model 1: no adjustment; Model 2: adjusted for age and sex; Model 3: adjusted for age, sex, smoking, working, educational level, living status, Hypertension, diabetes, cerebrovascular disease, mean arterial pressure, waist-hip circumference ratio, serum urid acid, iPTH, and hs-CRP levels.

### Non-linear relationships of serum magnesium level with MCI

**Figure 2.**
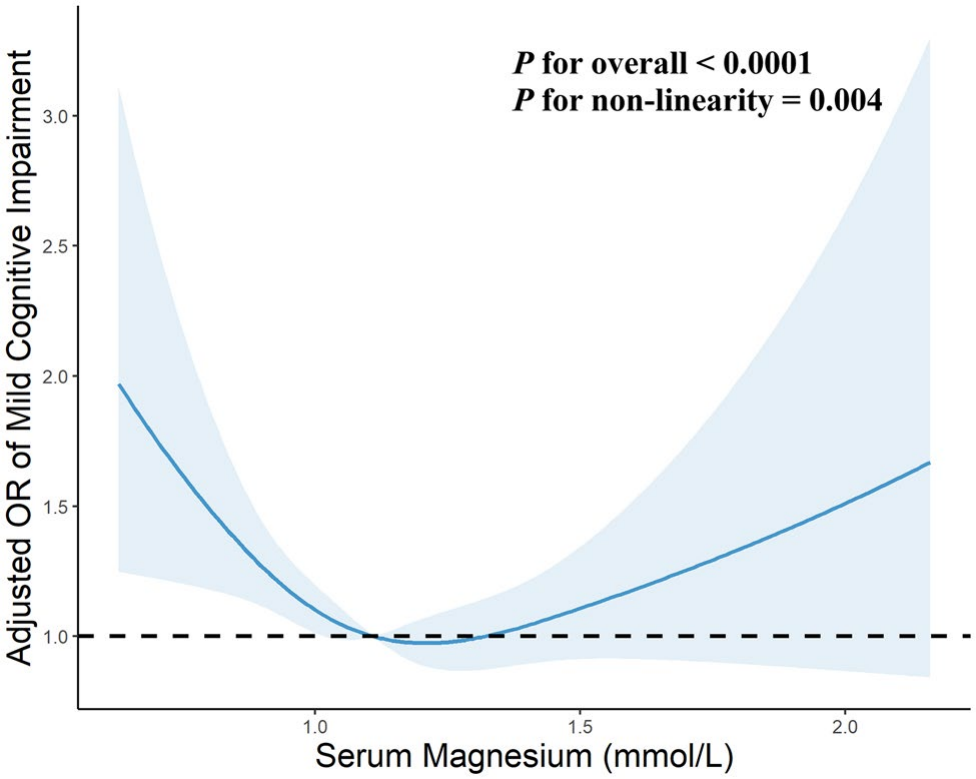
**Restricted cubic spline analysis for association of serum magnesium with the risk of mild cognitive impairment in hemodialysis patients.** Point estimates (blue solid line) and 95% confidence intervals (blue dashed area) were estimated by restricted cubic splines analysis with knots placed at the 10th, 50th, and 90th percentile. Model was adjusted for age, sex, smoking, working, educational level, living status, hypertension, diabetes, cerebrovascular disease, mean arterial pressure, waist-hip circumference ratio, serum urid acid, iPTH, and hs-CRP levels. OR, odds ratio.

As shown in Figure 2, the multivariate-adjusted RCS curves displayed that the relationships of serum magnesium level with MCI was U-shaped (*P* for likelihood ratio test = 0.004). Low and high serum magnesium levels were both associated with the risk of MCI. The threshold range of serum magnesium related to the lowest risk in multivariable-adjusted analyses was 1.12–1.24 mmol/L. As shown in Table 3, below the threshold, the risk of MCI was significantly decreased with per SD increment of magnesium level with ORs of 0.76 (95%CI: 0.62–0.93). Inversely, above the threshold, the risk of MCI was significantly increased with per SD increment of serum magnesium level (OR = 1.21, 95%CI: 1.02–1.43).

**Table 3.** Threshold effect analyses of serum magnesium level on MCI using two piecewise regression models.

Turnpoint	Model 1		Model 2		Model 3	
	OR (95%CI)	*P*-value	OR (95%CI)	*P*-value	OR (95%CI)	*P*-value
Magnesium <1.12 mmol/L (Per 1 SD)	0.72 (0.59–0.87)	0.001	0.75 (0.62–0.91)	0.004	0.76 (0.62–0.93)	0.007
Magnesium >1.24 mmol/L (Per 1 SD)	1.22 (1.03–1.45)	0.020	1.20 (1.01–1.43)	0.041	1.21 (1.02–1.43)	0.032

Note: *P* < 0.05 was considered statistically significant. Abbreviations: HD: hemodialysis; OR: odds ratio; CI: confidence interval; MCI: mild cognitive impairment; SD: standard deviation. Model 1: no adjustment; Model 2: adjusted for age and sex; Model 3: adjusted for age, sex, smoking, working, educational level, living status, Hypertension, diabetes, cerebrovascular disease, mean arterial pressure, waist-hip circumference ratio, serum urid acid, iPTH, and hs-CRP levels.

### Subgroup analyses of the risk of MCI

**Figure 3.**
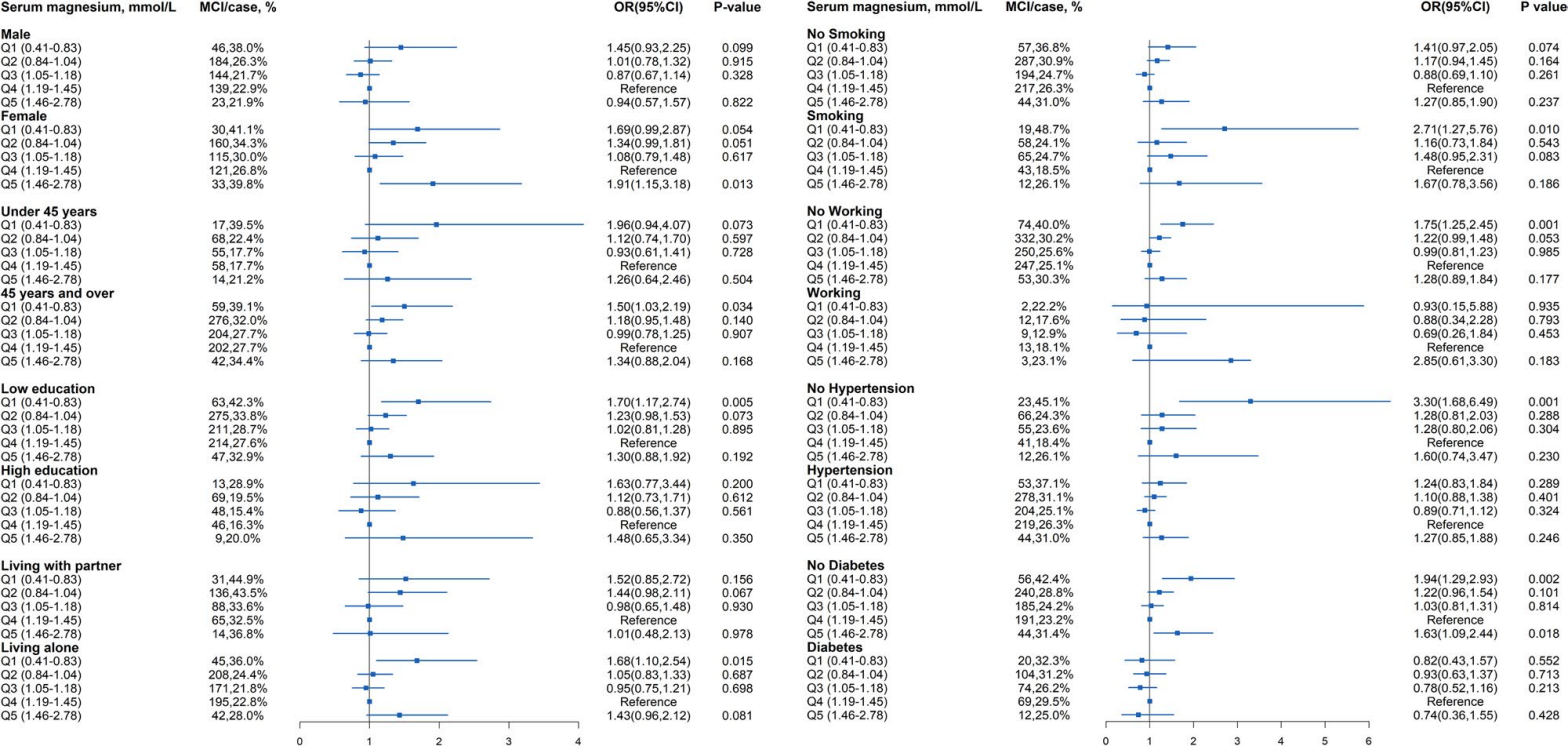
**Subgroup analyses for serum magnesium level Predicting mild cognitive impairment in hemodialysis patients.** Model was adjusted for age, sex, smoking, working, educational level, living status, hypertension, diabetes, cerebrovascular disease, mean arterial pressure, waist-hip circumference ratio, serum urid acid, iPTH, and hs-CRP levels. MCI, mild cognitive impairment; OR, odds ratio; CI: confidence interval.

The stratified analyses are demonstrated Figure 3 (Detail data as shown in Table S1). The serum magnesium level of 0.41-0.83 mmol/L in an increased risk of MCI were 1.50-fold for aged ≥ 45 years (*P* = 0.034), 1.70-fold for low education (*P* = 0.005), 1.68-fold for living alone (*P* = 0.015), 2.71-fold for smoking (*P* = 0.010), 1.75-fold for no working (*P* = 0.001), 3.30-fold for no hypertension (*P* = 0.001), and 1.94-fold for no diabetes (*P* = 0.002).

## Discussion

To the best of our knowledge, this is the first study to evaluate the association between serum magnesium levels and incident MCI among HD patients from multiple dialysis centers of southwestern China. We found that both low and high serum magnesium levels were significantly associated with an increased risk of MCI among HD patients. A U-shaped relationship was identified between serum magnesium and MCI, and the optimal serum magnesium range with the lowest risk of MCI was 1.12–1.24 mmol/L for this population. The risk of MCI decreased by 24% per SD increase in serum magnesium when serum magnesium was lower than 1.12 mmol/L, while a rise per SD increased the risk of MCI by 21% when the serum magnesium level exceeds 1.24 mmol/L.

In this study, the mean MMSE score was 27.5 and the prevalence of MCI was among 3652 HD patients. A meta-analysis including 42 studies of 3522 HD patients demonstrated that the estimated MMSE score was 27.1 [2], which was similar to that assessed in this study. A multicenter study from 11 dialysis centers in Beijing found that 37.8% of 613 Chinese HD patients had MCI [6]. A higher prevalence of MCI diagnosed with Montreal Cognitive Assessment was reported in a cross-sectional study from East China (51.6%) [19]. While, another study based on 616 Chinese HD patients reported 14.4% had MCI diagnosed with MMSE criteria [20]. The clinical heterogeneity of prevalence mainly depended on the differences in population demographics, sample sizes, diagnostic criteria, and measurements for the assessment for cognitive impairment. Nevertheless, the prevalence found in this study is comparable to many of the earlier results [3–6,19,20].

One of our major findings is that both low and high serum magnesium level is closely contributed to a higher risk of MCI in HD patients, and indicated a U-shaped association. This result is consistent with a previous study [15,21]. In the prospective population-based study, Kieboom et al. found that both low serum magnesium levels (≤0.79 mmol/L) and high serum magnesium levels (≥0.90 mmol/L) were associated with an increased risk of dementia during a median follow-up of 7.8 years among 9569 participants [15]. A large-scale multicenter study demonstrated that low and high magnesium concentrations were also associated with a high risk of vascular-related non-Alzheimer dementia, with the lowest risk observed at a concentration of 0.85 mmol/L, but no association was observed for Alzheimer’s dementia [21]. However, most previous studies have reported associations between cognitive impairment and low magnesium in a single direction of abnormality [12–14,22]. A cross-sectional study based on 1000 Qatari participants demonstrated that serum magnesium concentration was inversely associated with cognitive function measured by the mean reactive time [12]. A large, community-based cohort study consisting of 12040 participants found that low levels of serum magnesium were associated with an elevated risk of incident dementia, with a 24% increased risk for participants in the bottom compared to the top magnesium quintile during a 24.2-year follow-up period (HR 1.24, 95%CI 1.07–1.44) [13]. Tu et al. found that low level of serum magnesium (≤0.82 mmol/L) was independently associated with the occurrence of cognitive impairment at 1-month poststroke among acute ischemic stroke patients (OR 2.24, 95%CI 1.23–4.06) [22]. In addition, magnesium intake has also been proven to associate with better cognitive functioning and a decreased risk of developing cognitive impairment in several population-based cohort studies [23–24]. While, there is no still studies focused on the dialysis population. This study demonstrated that patients with lower magnesium levels had a 24% increased risk of incident MCI among HD patients, which is an excellent extension of the previous studies.

Although the disparity in the study population, in accordance with our results, several previous studies have observed the U-shaped association between magnesium levels and other chronic inflammatory conditions [25–29]. A prospective study containing 5044 Chinese adults showed a U-shaped association between serum magnesium level and insulin resistance, and type 2 diabetes, with low and high levels associated with increased risk [25]. Yue et al. found a U-shaped relationship between serum magnesium and 28-day in-hospital all-cause mortality in critically ill children admitted to the pediatric intensive care unit with 0.74–0.93 mmol/L as the optimal serum magnesium range for the lowest risk of mortality [26]. The similar U-shaped relationships between serum magnesium and mortality no matter in traumatic brain injury patients, kidney transplant recipients or coronary artery disease patients have also been proven in previous studies [27–29]. In this study, we found that the optimal serum magnesium threshold range for the lowest risk of MCI was 1.12–1.24 mmol/L. Thus, magnesium supplementation should be especially used with caution in this specific population. To our best knowledge, this is the first time to evaluate the non-linear association between serum magnesium and cognitive impairment among HD patients.

The underlying mechanism of the U-shaped association is still not clear. For the association between low magnesium and MCI, some potential mechanisms have been reported. First, neuronal magnesium plays critical roles in multiple biochemical processes involved in cognitive functions, including cell membrane stability and integrity, N-methyl-D-aspartate (NMDA)-receptor response to excitatory stimuli, and Ca-antagonist action [8,30]. Magnesium deficiency will disturb these above processes, leading to cognitive impairment. Second, insufficient magnesium triggers oxidate stress through stimulating the secretion of various inflammatory mediators, accelerating neurodegeneration [31,32]. Additionally, hypermagnesemia can cause neuromuscular toxicity, which impairs cognitive function [33]. High magnesium has also effects on cellular electrical conduction and vasodilation, leads to hypotension, which could be reflected in cognitive impairment [34]. No matter how more studies are needed to clarify the exact mechanism of the U-shaped association.

The advantage of the present study lies in its relatively large sample size and multicenter study design. Regarding clinical importance, our novel findings are conducive to understanding the risk stratification of magnesium and remind us that when initiating magnesium-supplement therapy in clinical practice, and attention should be paid to assessing the absolute risk of cognitive impairment, rather than starting treatment based solely on a moderate increase in levels of a specific magnesium marker. Anyway, there are still some limitations to this study. First, cognitive function was only measured with the MMSE score, which has relatively low sensitivity for the detection of mild and early cognitive impairment. In addition, MMSE can be highly influenced by an individual’s level of education, leading to a bias against people with poor educational levels. However, to our data, current various studies have evaluated cognitive impairment with MMSE score, due to its simplicity and operability. Second, in this cross-sectional study, although we adjusted many relevant confounding variables that were considered to influence cognitive function, residual confounders, and hidden comorbidities might have been not eliminated, such as functional status. Third, the data about daily magnesium consumption was not obtained and was not adjusted in these subsequent analyses. Finally, our study was performed in a representative sample of HD patients in the province of Southwestern China, so our results may not be easily extrapolated to the population in other regions.

In summary, this study demonstrated that serum magnesium had a U-shaped association with cognitive impairment among HD patients. Both low and high serum magnesium can increase the risk of cognitive impairment. The optimal serum magnesium range with the lowest risk of cognitive impairment was 1.12–2.24 mmol/L. Proper attention should be paid to addressing the abnormal magnesium status of HD patients in clinical practice for the improvement of cognitive function. Future studies targeted the association between serum magnesium and cognitive impairment in HD patients are essential.

## Supplementary Material

Supplemental Material

## Data Availability

The data presented in this study are available from the corresponding author on reasonable request.
