# Supplementary material for: U-shaped association of serum magnesium with mild cognitive impairment among hemodialysis patients: a multicenter study
Source: Ren Fail. 2023 Jul 10;45(1):2231084. doi: 10.1080/0886022X.2023.2231084 (PMC10334860; doi:10.1080/0886022X.2023.2231084)
Supplement: Supplemental Material [file IRNF_A_2231084_SM6260.pdf]

Table S1. Subgroup analyses of association between serum magnesium levels and mild cognitive impairment among HD patients

Serum magnesium (mmol/L)	MCI* OR(95%CI)	MCI* *P* value	Serum magnesium (mmol/L)	MCI* OR(95%CI)	MCI* *P* value
**Male**			**No Smoking**		
Q1 (0.41-0.83)	1.45(0.93,2.25)	0.099	Q1 (0.41-0.83)	1.41(0.97,2.05)	0.074
Q2 (0.84-1.04)	1.01(0.78,1.32)	0.915	Q2 (0.84-1.04)	1.17(0.94,1.45)	0.164
Q3 (1.05-1.18)	0.87(0.67,1.14)	0.328	Q3 (1.05-1.18)	0.88(0.69,1.10)	0.261
Q4 (1.19-1.45)	Reference		Q4 (1.19-1.45)	Reference	
Q5 (1.46-2.78)	0.94(0.57,1.57)	0.822	Q5 (1.46-2.78)	1.27(0.85,1.90)	0.237
**Female**			**Smoking**		
Q1 (0.41-0.83)	1.69(0.99,2.87)	0.054	Q1 (0.41-0.83)	2.71(1.27,5.76)	0.010
Q2 (0.84-1.04)	1.34(0.99,1.81)	0.051	Q2 (0.84-1.04)	1.16(0.73,1.84)	0.543
Q3 (1.05-1.18)	1.08(0.79,1.48)	0.617	Q3 (1.05-1.18)	1.48(0.95,2.31)	0.083
Q4 (1.19-1.45)	Reference		Q4 (1.19-1.45)	Reference	
Q5 (1.46-2.78)	1.91(1.15,3.18)	0.013	Q5 (1.46-2.78)	1.67(0.78,3.56)	0.186
**Aged<45 years**			**No Working**		
Q1 (0.41-0.83)	1.96(0.94,4.07)	0.073	Q1 (0.41-0.83)	1.75(1.25,2.45)	0.001
Q2 (0.84-1.04)	1.12(0.74,1.70)	0.597	Q2 (0.84-1.04)	1.22(0.99,1.48)	0.053
Q3 (1.05-1.18)	0.93(0.61,1.41)	0.728	Q3 (1.05-1.18)	0.99(0.81,1.23)	0.985
Q4 (1.19-1.45)	Reference		Q4 (1.19-1.45)	Reference	
Q5 (1.46-2.78)	1.26(0.64,2.46)	0.504	Q5 (1.46-2.78)	1.28(0.89,1.84)	0.177
**Age≥45 years**			**Working**		
Q1 (0.41-0.83)	1.50(1.03,2.19)	0.034	Q1 (0.41-0.83)	0.93(0.15,5.88)	0.935
Q2 (0.84-1.04)	1.18(0.95,1.48)	0.140	Q2 (0.84-1.04)	0.88(0.34,2.28)	0.793
Q3 (1.05-1.18)	0.99(0.78,1.25)	0.907	Q3 (1.05-1.18)	0.69(0.26,1.84)	0.453
Q4 (1.19-1.45)	Reference		Q4 (1.19-1.45)	Reference	

Information Classification: General

Q5 (1.46-2.78)	1.34(0.88,2.04)	0.168	Q5 (1.46-2.78)	2.85(0.61,3.30)	0.183
**Low education**			**No Hypertension**		
Q1 (0.41-0.83)	1.70(1.17,2.74)	0.005	Q1 (0.41-0.83)	3.30(1.68,6.49)	0.001
Q2 (0.84-1.04)	1.23(0.98,1.53)	0.073	Q2 (0.84-1.04)	1.28(0.81,2.03)	0.288
Q3 (1.05-1.18)	1.02(0.81,1.28)	0.895	Q3 (1.05-1.18)	1.28(0.80,2.06)	0.304
Q4 (1.19-1.45)	Reference		Q4 (1.19-1.45)	Reference	
Q5 (1.46-2.78)	1.30(0.88,1.92)	0.192	Q5 (1.46-2.78)	1.60(0.74,3.47)	0.230
**High education**			**Hypertension**		
Q1 (0.41-0.83)	1.63(0.77,3.44)	0.200	Q1 (0.41-0.83)	1.24(0.83,1.84)	0.289
Q2 (0.84-1.04)	1.12(0.73,1.71)	0.612	Q2 (0.84-1.04)	1.10(0.88,1.38)	0.401
Q3 (1.05-1.18)	0.88(0.56,1.37)	0.561	Q3 (1.05-1.18)	0.89(0.71,1.12)	0.324
Q4 (1.19-1.45)	Reference		Q4 (1.19-1.45)	Reference	
Q5 (1.46-2.78)	1.48(0.65,3.34)	0.350	Q5 (1.46-2.78)	1.27(0.85,1.88)	0.246
**Living with partner**			**No Diabetes**		
Q1 (0.41-0.83)	1.52(0.85,2.72)	0.156	Q1 (0.41-0.83)	1.94(1.29,2.93)	0.002
Q2 (0.84-1.04)	1.44(0.98,2.11)	0.067	Q2 (0.84-1.04)	1.22(0.96,1.54)	0.101
Q3 (1.05-1.18)	0.98(0.65,1.48)	0.930	Q3 (1.05-1.18)	1.03(0.81,1.31)	0.814
Q4 (1.19-1.45)	Reference		Q4 (1.19-1.45)	Reference	
Q5 (1.46-2.78)	1.01(0.48,2.13)	0.978	Q5 (1.46-2.78)	1.63(1.09,2.44)	0.018
**Living alone**			**Diabetes**		
Q1 (0.41-0.83)	1.68(1.10,2.54)	0.015	Q1 (0.41-0.83)	0.82(0.43,1.57)	0.552
Q2 (0.84-1.04)	1.05(0.83,1.33)	0.687	Q2 (0.84-1.04)	0.93(0.63,1.37)	0.713
Q3 (1.05-1.18)	0.95(0.75,1.21)	0.698	Q3 (1.05-1.18)	0.78(0.52,1.16)	0.213
Q4 (1.19-1.45)	Reference		Q4 (1.19-1.45)	Reference	
Q5 (1.46-2.78)	1.43(0.96,2.12)	0.081	Q5 (1.46-2.78)	0.74(0.36,1.55)	0.428

Note: $P<0.05$ was considered statistically significant. Abbreviations: HD, hemodialysis; OR, odds ratio; CI, confidence interval; MCI, mild cognitive

Information Classification: General

impairment. *adjusted for age, sex, smoking, working, educational level, living status, Hypertension, diabetes, cerebrovascular disease, mean arterial pressure, waist-hip circumference ratio, serum urid acid, iPTH, and hs-CRP levels.

Information Classification: General
